# Choice of Antimicrobials in Surgical Prophylaxis - Overuse and Surgical Site Infection Outcomes from a Tertiary-Level Care Hospital

**DOI:** 10.3389/fphar.2022.849044

**Published:** 2022-04-11

**Authors:** Prasanna Vippadapu, Syed Wasif Gillani, Dixon Thomas, Fiaz Ahmed, Shabaz Mohiuddin Gulam, Rana Kamran Mahmood, Vineetha Menon, Semira Abdi, Hassaan Anwer Rathore

**Affiliations:** ^1^ College of Pharmacy, Gulf Medical University, Ajman, United Arab Emirates; ^2^ Thumbay University Hospital, Ajman, United Arab Emirates; ^3^ ResponsePlus Medical, Abu Dhabi, United Arab Emirates; ^4^ Dubai Pharmacy College, Dubai, United Arab Emirates; ^5^ College of Pharmacy, Qatar University, Doha, Qatar

**Keywords:** surgical antimicrobial prophylaxis, ceftriaxone, cefotaxime, surgeries, surgical site infections, antimicrobial stewardship

## Abstract

**Background:** This study was aimed to describe the choice of Surgical Antimicrobial Prophylaxis at a tertiary-level care hospital in United Arab Emirates. It also associated the choice between two leading antimicrobials for the SAP to the site of surgery.

**Methods:** A descriptive drug use evaluation was performed retrospectively to study choices of antimicrobials in surgical antibiotic prophylaxis. An analytical cross-sectional study design was used to develop a hypothesis regarding the choice of ceftriaxone. Data were collected from the medical records of Hospital from July 2020 to December 2020. Results were presented in numbers and percentages.

**Results:** SAP data were collected from 199 patients, of which 159 were clean or clean-contaminated. Dirty surgeries (18) needed a higher level of antimicrobials as there were infections to be treated. For other surgeries with no infection, overuse of antimicrobials was found regarding the choice of antimicrobials. Surgical antibiotic Prophylaxis was administered within the recommended time prior to surgeries. Ceftriaxone was preferred over cefuroxime in all types of surgeries based on the timing of Surgical Antibiotic Prophylaxis, wound classification, and the surgical site. A statistically significant association for choice of ceftriaxone over cefuroxime was found regarding surgical sites (p-value <0.05). About 99% of the patients were prescribed discharge antimicrobials when 158 (80%) surgeries were clean or clean-contaminated.

**Conclusion:** Overuse of antimicrobials was found in surgical antimicrobial prophylaxis. Ceftriaxone was preferred more than cefuroxime in all types of surgeries. No surgical site infections were reported. A follow-up comparative study is recommended to decrease antimicrobial use without increasing risk of surgical site infection.

## Introduction

The surgical antimicrobial prophylaxis (SAP) often seems to have overuse of antimicrobials in the effort to prevent surgical site infections (SSI) (Barie, 2013). It is important to avoid overuse of antimicrobials, control the risk of microbial resistance and prevent SSIs by optimal use of antimicrobials in SAP. One of the most comprehensive SAP guidelines was developed jointly by the American Society of Health-System Pharmacists (ASHP), the Infectious Diseases Society of America (IDSA), the Surgical Infection Society (SIS), and the Society for Healthcare Epidemiology of America (SHEA). For convenience, this guideline is hereafter mentioned as ASHP guidelines. The ASHP guidelines of SAP provide comprehensive details in indication, selection, dosing, duration, and the timing of antimicrobials (Bratzler et al., 2013). The guideline-directed SAP has a beneficial effect (if properly implemented) to ensure that appropriate antimicrobials are chosen to cover the most likely pathogen at the correct dose. Evidence suggests that the administration of antimicrobials before surgery prevents infection after surgery, thus reducing SSI (Barie, 2013; Bratzler et al., 2013).

Antimicrobial stewardship program (ASP) plays a significant role in the empirical, prophylactic, and therapeutic use of antimicrobials (Bratzler et al., 2013). SSIs are associated with morbidity, mortality, increased healthcare costs, and hospital readmissions. A detailed understanding of factors and strategies is needed to optimize SAP and patient care (Alemkere, 2018). Prolonged use of antimicrobials can increase the risk of adverse events such as *Clostridium difficile* infections, antimicrobial resistance, acute kidney injury, and many other safety concerns. The SAP interventions should target before, while, and after surgery as needed (Abubakar et al., 2019). Several studies suggested that guideline compliance to SAP was compromised as there was a gap in awareness, availability of guidelines to health care professionals, acceptance of guidelines by surgeons, and lack of clarity about roles and responsibilities (Abubakar et al., 2019; Surgical Site Infection (SSI) Event, 2010). A well-implemented evidence-based (international/local) ASP program is essential to improve the appropriate use of SAP.

Surgical wound classication is categorized by the degree of gross contamination (clean, clean-contaminated, contaminated, and dirty) and is used in conjunction with American Society of Anesthesiology and procedure duration to find out the risk of SSIs (Surgical Site Infection, 2010). Many studies showed the use of different antimicrobial agents despite the guidelines recommendations, even not following the local/internal guidelines. Many factors could contribute to deviations from international guidelines or even local/internal guidelines that aremedication availability (shortage of medicine that need to be given as a prophylaxis as per the guidelines), less trust in local practice guidelines, healthcare delivery policies, personal experiences, and antimicrobial resistance in a particular setting (Cohen et al., 2017; Segala et al., 2020).

Researchers suggested in a study that around 55% of surgical site infections may be preventable with the help of evidence based strategies implementation appropriately (Umscheid et al., 2011). Therefore, evaluating multiple factors related to SAP is essential to reduce SSI rates and healthcare costs with better patient outcomes (Cheng et al., 2015).

As the choice of antimicrobials in SAP depends on many factors, describing the SAP practices at that hospitalis essential. Evaluating the choices of antimicrobials in SAP helps explore areas of improvement. Furthermore, it is helpful to compare good practices in other hospitals and optimize antimicrobial use as the threat of antimicrobial resistance is a global problem and achieving better patient outcomes is a local need. In this study, we aim to describe the choices of antimicrobials in SAP. It was also necessary to generate a hypothesis for further testing if ceftriaxone is preferred over cefuroxime in surgical antimicrobial prophylaxis. The objective of the study is to evaluate the choice of surgical antimicrobial prophylaxis at a tertiary-level care hospital in United Arab Emirates. Also, to find out the choice of SAP is ceftriaxone or cefuroxime.

## Materials and Methods

### Study Design

The study design was a retrospective, descriptive antimicrobial use evaluation among patients who underwent a surgical procedure in a tertiary-level care hospital in the UAE. It was basically an observational study. For exploring the significance of choosing ceftriaxone, an analytical cross-sectional design was used.

### Study Population

Data of all the patients who underwent a surgical procedure at the hospital from July 2020 to December 2020 were collected considering inclusion and exclusion criteria. The clinical pharmacists at the hospital informed that over 100 surgeries being conducted in 6 months. Thus, data were collected for 6 months to get minimum sample of 100 surgeries.

### Inclusion and Exclusion Criteria

Patients who are above 18 years and underwent any surgical procedures such as (ENT, Thyroid, orthopedic, gastrointestinal, urology, breast and miscellaneous surgeries) were included in the study. However, dental procedures and non-surgical medical procedures were excluded. Surgeries in special populations, pediatric, geriatric (patients above 85 years old), pregnant, cancer or transplant and immunocompromised patients were excluded from this study.

### Study Settings

The study was conducted in a teriary-level care hospital in the UAE. The hospital treated patients in multiple specialties with inpatient and outpatient services and it have an operation theater complex. Main surgeries happening at the hospital included cesarean sessions, general surgeries, orthopedic, neurological, cardiac, and other surgeries. Transplant surgeries were not performed at the study site, but other surgeries are performed on patients who have a transplant.

### Data Collection and Analysis

All patient data were collected from the medical records through electronic system. The research student collected the data. ASHP Antimicrobial Stewardship Program guidelines were used to compare the ASP use in our study (Bratzler et al., 2013) in addition to the Hospital ASP guidelines. In addition, socio-demographic characteristics of the patients, surgery-related information (site of surgery, duration of surgery, previous history of surgery, surgery type, hospital stay after surgery, wound class and occurrence of SSI after surgery within 30 days), and antimicrobials used (preoperative, postoperative and discharge) were collected.

### Statistical Analysis

The statistical package of social sciences (SPSS version 26.0) was used to analyze the data. Pearson chi-square test was used to find an association between the timing of antimicrobial administration, or type of surgical site to the choice of ceftriaxone or cefuroxime. P-values less than 0.05 were considered statistically significant. The test was performed to generate a hypothesis, not to prove the hypothesis. Sample size needs to be calculated for analytical cross-sectional studies in the future.

### Ethics Approval

The study was approved by the institutional review board (IRB). Permission from the Medical Director was obtained with a copy of IRB approval for data collection. Patient confidentiality has been maintained. While processing the data, patient identifiers were removed.

## Results and Findings

### Patient Demographics

Data were collected from the electronic medical records of 199 patients after considering inclusion and exclusion criteria. There were no more than one surgery in single patient. Pregnancy-related surgeries were a major group of surgeries that were not included in this study. All the study population underwent surgery at Hospital. Their demographic details are shown in Table 1. The majority were adult males with a Body Mass Index (BMI) of normal or overweight categories.

**TABLE 1:** Demographics of the study population.

Characteristics	Number	Percentage (%)
Age (yrs)		
18 to 65	193	96.9
66 to 80	6	3.0
Gender		
Male	134	67.3
Female	65	32.7
BMI (kg/m^2^)		
Underweight (<18.5)	2	1.0
Normal (18.5–24.9)	52	26.1
Overweight (25–29.9)	84	42.2
Obese (30–34.9)	41	20.6
Severely obese (35–39.9)	8	4.0
Morbid obese (40 and above)	12	6.0
Allergies		
Penicillin allergy	2	1
Sulpha drugs allergy	1	0.5
None	196	98.5
Medical history		
Hypertension	9	4.5
Diabetes and hyperlipidemia and thyroid	6	3.0
Diabetes	5	2.5
Hypothyroidism	5	2.5
Hypertension, hyperlipidemia and diabetes	4	2.0
Hyperlipidemia	1	0.5
Depression	1	0.5
Cardiovascular disease and hyperlipidemia	1	0.5

BMI, body mass index.

### Antimicrobial Administration Timing Before Surgery

**FIGURE 1:**
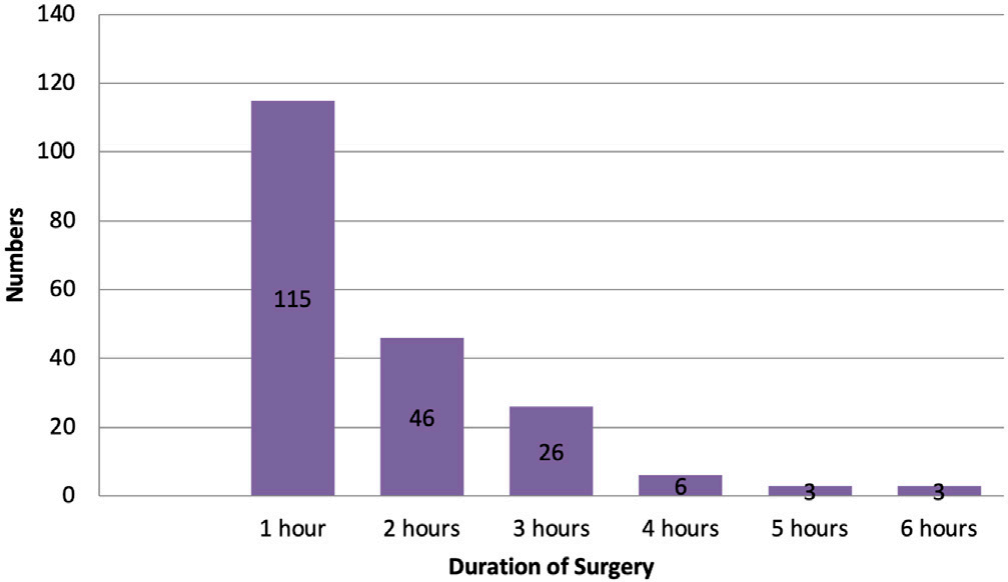
Duration of surgeries among the study population.

The majority of surgeries were performed within an hour, as shown in Figure 1. Only a few surgeries lasted more than 3 h. For surgeries happening more than 3 or 4 h, ASHP guidelines recommend an intraoperative dose of antimicrobials depending on the type of antimicrobials used. For example, cefuroxime provides prophylactic coverage for 4 h, but cefotaxime needs to be administered intraoperatively if surgery prolongs over 3 h. Unfortunately, data regarding the intraoperative administration of antimicrobials were not available to the research team. (Bratzler et al., 2013).

Depending on the site of surgery, as shown in Table 2, there were some differences when surgical antimicrobials were administered. On average, the timing of administration of antimicrobials prior to surgery was similar and within the recommended time. The ASHP guidelines recommend administering SAP 60–120 min prior to surgery, with some exceptions (Bratzler et al., 2013). There were two surgeries; calculous of kidney surgery (135 min) and anterior cervical discectomy and fusion (150 min) when an SAP was administered over 120 min prior to surgery (this is mentioned in miscellaneous surgeries in Table 2).

**TABLE 2:** Surgical site and antimicrobial administration time.

Type of surgery based on site	Time of antimicrobial given prior to surgery in minutes	Average antimicrobial administration time prior to surgery in minutes
Ear Nose Throat Surgeries	25 to 100	69.1
Thyroid surgeries	60 to 70	67.5
Orthopedic surgeries	15 to 115	66.7
Gastrointestinal surgeries	20 to 120	64.2
Urologic surgeries	30 to 120	62.6
Surgeries related to breast	25 to 100	58.6
Miscellaneous	20 to 150	58

Ceftriaxone and cefuroxime were the most commonly used antimicrobials in the study population. Pre-surgical administration of ceftriaxone and cefuroxime were all within 2 h prior to surgery (Table 2).

### Surgeries Based on Wound Classification

**FIGURE 2:**
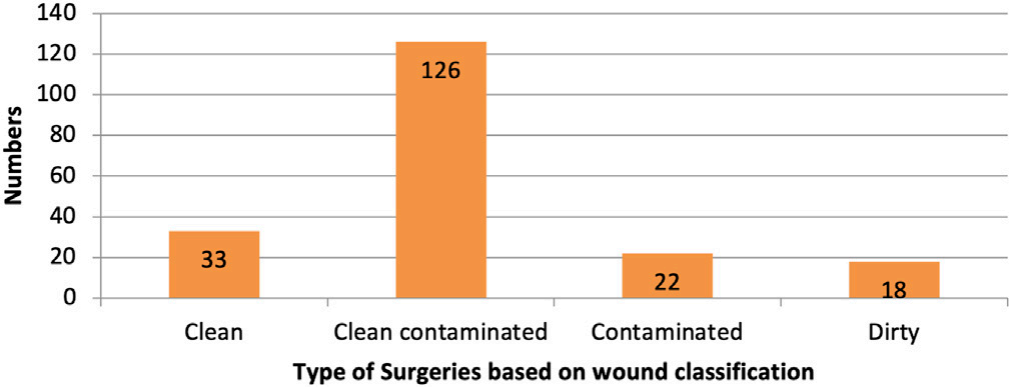
Type of surgeries based on wound classification.

The majority of surgeries were clean-contaminated, followed by clean, contaminated, and dirty, as shown in Figure 2.

Dirty surgeries with infection needed antimicrobials for the treatment of existing infections and prevention of further infections. The use of higher antimicrobials was justified in dirty surgeries. There is a higher risk for SSIs in contaminated surgeries that might motivate surgeons to prescribe higher antimicrobials. Table 3 shows pre-surgical antimicrobials administered based on wound classification.

**TABLE 3:** Surgical wound classification and antimicrobials administered.

Type of surgeries based on wound classification	Ceftriaxone	Ceftriaxone + metronidazole	Cefuroxime	Cefepime	Amikacin	SAP not given	Miscellaneous
Clean (33)	16 (48%)	2 (6%)	1 (3%)	3 (9%)	1 (3%)	4 (12%)	6 (18%)
Clean contaminated (126)	54 (43%)	5 (4%)	22 (17%)	9 (7%)	8 (6%)	15 (12%)	13 (10%)
Contaminated (22)	8 (36%)	8 (36%)	3 (14%)	1 (5%)	-	2 (9%)	-
Dirty (18)	8 (44%)	4 (22%)	1 (6%)	2 (11%)	-	-	3 (17%)
Total (199)	86	19	27	15	9	21	22

SAP, surgical antimicrobial prophylaxis.

There were not enough data to run the Pearson Chi-Square or Fisher’s extract test to find an association between ceftriaxone or cefuroxime with the type of surgeries based on wound classification. However, the preference in numbers and percentages was for ceftriaxone, as shown in Table 4.

**TABLE 4:** Pre-surgical use of ceftriaxone and cefuroxime based on wound classification.

Surgery type based on wound classification	Ceftriaxone	Cefuroxime
Clean (17)	16 (94%)	1 (6%)
Clean contaminated (76)	54 (71%)	22 (29%)
Contaminated (11)	8 (73%)	3 (27%)
Dirty (9)	8 (89%)	1 (11%)
Total	86	27

## Surgeries Based on Surgical Site

Deviations were observed to SAP at the study site compared to the ASHP SAP guidelines. More information is provided in Table 5.

**TABLE 5:** Surgical site and antimicrobials administered compared with guidelines.

Type of surgery (site)	Number (%)	ASHP guideline recommendation	Pre and post SAP
Gastrointestinal Surgeries	73 (36.6%)	Cefazolin	Ceftriaxone 31 (42.4%)
		Cefazolin + metronidazole	Ceftriaxone + metronidazole 16 (21.9%)
		Cefoxitin	Cefepime 12 (16.4%)
		Cefotetan	Metronidazole 3 (4.1%)
		Ceftriaxone + metronidazole	Ciprofloxacin 2 (2.7%)
			Cefuroxime 1 (1.3%)
			Ciprofloxacin + metronidazole 1 (1.3%)
			Cefepime + metronidazole 1 (1.3%)
			SAP not given 6 (8.2%)
Orthopedic surgeries	41 (20.6%)	Cefazolin cefuroxime	Ceftriaxone 24 (58.5%)
			Cefuroxime 14 (34.1%)
			Ceftriaxone + metronidazole 1 (2.4%)
			SAP not given 2 (4.8%)
ENT surgeries	25 (12.5%)	Cefazolin	Ceftriaxone 11 (44%)
		Amoxicillin sulbactam	Cefuroxime 10 (40%)
		Amoxicillin clavulanate clindamycin	Amoxicillin clavulanate 1 (4%)
			Cefepime 1 (4%)
			SAP not given 2 (8%)
Thyroid surgery	2 (1%)	Amoxicillin clavulanate	Ceftriaxone 1 (50%)
			Cefepime 1 (50%)
Urologic surgeries	36 (18%)	Cefazolin	Ceftriaxone 12 (33.3%)
		Trimethoprim-sulfamethoxazole	Amikacin 9 (25%)
		Fluoroquinolones	Cefotaxime 4 (11.1%)
			Gentamicin 2 (5.5%)
			Cefuroxime 2 (5.5%)
			Ciprofloxacin 1 (2.7%)
			SAP not given 6 (16.6%)
Surgeries related to breast	11 (5.5%)	Cefazolin	Ceftriaxone 3 (27.2%)
		Ampicillin sulbactam	Ceftriaxone + metronidazole 1 (9%)
		Clindamycin	Moxifloxacin + metronidazole 1 (9%)
		Vancomycin	Cefepime + metronidazole 1 (9%)
			Ciprofloxacin + metronidazole 1 (9%)
			Moxifloxacin 1 (9%)
			Cefepime 1 (9%)
			SAP not given 2 (18.1%)
Miscellaneous	11 (5.5%)	Cefazolin	Ceftriaxone 4 (36.3%)
			Ceftriaxone + metronidazole 1 (9%)
			Amoxicillin clavulanate 1 (9%)
			Cefotaxime 1 (9%)
			Piperacillin tazobactam 1 (9%)
			SAP not given 3 (27.2%)

ASHP, American Society of Health-System Pharmacy; SAP, surgical antimicrobial prophylaxis.

Other than orthopedic and ear, nose, throat (ENT), surgeries were combined to control expected frequencies of <5 in <25% cells. The Pearson Chi-Square test showed a statistically significant association (p-value < 0.05) of choice of ceftriaxone over cefuroxime in surgeries (orthopedic, ENT, and others) categorized based on surgical site. Though the preference in choice was ceftriaxone in all surgeries, as shown in Table 6, it needs to be further evaluated after calculating the appropriate sample size. Among cefuroxime use, orthopedic and ENT surgeries showed relatively higher prescribing. This shows that ASP programs shall initially focus on these two types of surgeries to improve the use of cefuroxime.

**TABLE 6:** Pre-surgical use of ceftriaxone and cefuroxime based on the type of surgery site.

Sl	Type of surgery based on site	Ceftriaxone	Cefuroxime	Association
1	Orthopedic surgeries (38)	24 (63%)	14 (37%)	P-value <0.05^a^
2	ENT (ear nose throat) surgeries (21)	11 (52%)	10 (48%)	
3	Others (54)	51 (94%)	3 (6%)	
	Total	86	27	

a Pearson Chi-Square test showed association, need to prove it with a bigger study.

In the case of clean and clean-contaminated surgeries, it is recommended not to prescribe antimicrobials at discharge from the hospital. However, almost all patients received discharge antimicrobials (99%) when it might not be required for clean and clean-contaminated surgeries, 159 (80%). Ceftriaxone and gentamicin are parenteral antimicrobials prescribed at discharge, probably for patients who have access to nursing care on a daily basis. Cefuroxime was more commonly used at discharge compared to ceftriaxone. More details are provided in table 7.

**TABLE 7:** Antimicrobials prescribed at post-surgical hospital discharge.

Antimicrobials given after surgery	Total N (%)
Ciprofloxacin + metronidazole	56 (28.1)
Cefuroxime	44 (22.1)
Ciprofloxacin	38 (19.0)
Cefdinir	23 (11.5)
Levofloxacin	11 (5.5)
Amoxicillin clavulanate	7 (3.5)
Moxifloxacin	5 (2.5)
Ceftriaxone	3 (1.5)
Cefdinir + metronidazole	2 (1.0)
Cefepime + metronidazole	1 (0.5)
Cefixime + metronidazole	1 (0.5)
Cefepime	1 (0.5)
Cefditoren	1 (0.5)
Cefpodoxime	1 (0.5)
Moxifloxacin + metronidazole	1 (0.5)
Gentamicin	1 (0.5)
Discharge antimicrobials not given	3 (1.5)
Total	199 (100%)

No SSIs were reported to any of the study population during the study period July 2020 to December 2020. As per ASP data of the hospital, from January 2021 to May 2021, there were no reports of any SSIs in any surgeries conducted. There was an SSI case in a pediatric patient in June 2021.

## Discussion

Our study population received SAP similar to some studies but different from the guidance of using first or second-generation cephalosporins (Umscheid et al., 2011; Cheng et al., 2015; Cohen et al., 2017; Halawi et al., 2018). The ASHP guidelines and SAP guidelines in the study setting were recommending first or second-generation cephalosporins. In all types of surgeries, ceftriaxone (third-generation cephalosporin) was used more than cefuroxime (second-generation cephalosporin).

In our study, the majority of the surgical procedures were clean-contaminated or clean based on wound classification. Similar findings were reported in other studies where clean-contaminated surgeries were higher, followed by clean surgeries. In addition, discharge from hospital antimicrobials was prescribed for most patients, including clean and clean-contaminated surgeries, when it was not necessary (Ozgun et al., 2010; Halawi et al., 2018).

In our study, antimicrobial administration time was reported <1 h in most surgeries, which was consistent with many studies. Some studies suggested no significant difference in SSI risk comparing 120–60 min *versus* 60–0 min before surgical procedure. Administration >120 min before incision shall increase SSIs risk (Alahmadi et al., 2020).

The SAP guidelines reported multifactorial contexts of SSIs, including drug selection, dose administration, time of administration, duration of surgery, patient-related factors like BMI. Several studies have linked antimicrobials’ pharmacokinetics, such as half-life, adequate tissue concentrations, to surgical site infections. Therefore, adherence to SAP guidelines varies considering multiple aspects (de Jonge et al., 2017; Siddiqi et al., 2019; Alahmadi et al., 2020). Further research is required to prove if there is any association exists.

Our results also show a difference in gender distribution who underwent surgeries. The duration of surgeries was significantly higher in females compared to males. As duration A similar finding was reported in many studies, but contrasting findings were reported in a study where males (51.2%) had a higher duration compared to females (48.8%) (Umscheid et al., 2011; Cheng et al., 2015; Halawi et al., 2018; Alahmadi et al., 2020). In our study number of males are significantly more than female that can impact the overall duration of antimicrobials.

For none of the surgeries, cefazolin was used. Cefazolin is recommended for SAP as per the guidelines. The use of narrow-spectrum antimicrobials needs to be avoided and to reduce or even stop in clean surgeries. Overuse of antimicrobials will increase resistance among microbes and may lead to the emergence of multi-drug resistant organisms (Mendelson and Matsoso, 2015; Mossanen et al., 2015; Hawn and Knowlton, 2019; Niraula et al., 2021). Several studies recommended monitoring hospital prescriptions that can help in guiding the antimicrobial use and resistance pattern among pathogens. (Lai et al., 2016; Tiri et al., 2020).

Antimicrobial administration time before surgical incision was as per the hospital guidelines (60–120 min). Therefore, it is essential to review the pattern of antimicrobial prophylaxis in relation to the duration of surgery (Mu et al., 2011; Argaw et al., 2017; Tiri et al., 2020). However, no data was found about intraoperative administration for the surgeries that took more than 3 h. As per the practitioners, intraoperative antimicrobial doses were administered when surgery was prolonged more than 3 h. The intraoperative dose depends on the type of antimicrobial, time of administration before surgery, duration of the surgical procedure, and surgical site (Mu et al., 2011; Argaw et al., 2017).

Studies show different choices of antimicrobials, e.g., in otolaryngological surgeries, cefazolin or ampicillin-sulbactam/amoxicillin-clavulanate or clindamycin were used (Ottoline et al., 2013). Most of the surgeries in our study population were clean-contaminated or clean; SAP might be unnecessary as per the ASHP guidelines for surgeries in some locations. However, the findings showed the overuse of antimicrobials. The results also reported no surgical site infection in any of the cases collected. In fact, from December 2020 to May 2021, no surgical site infection was reported to any patients treated at the study site. Many of the other studies were showing more SSIs from US (1.9%), Pakistan (9.29%), Ethiopia (20.6%), and as low as from 2.5 to 41.9% depending on hospital settings and the extent of implementation of sterilization and aseptic techniques. Limited resources at hospitals might also be factors contributing to SSIs (Linda, 2003; Mu et al., 2011; Tefera et al., 2020). It is understandable that choices of antimicrobials vary depending on multiple aspects. Studies related to the antimicrobials and stewardships are less evident in Asian countries.

Retrospective data collection on ASP is convenient and quick but will be affected by missing data issues. Some studies show missing data on intra-operative surgical antimicrobial prophylaxis. Deviation from ASP guidance is also reported in other studies. For example, in a study, more than 95% of surgeries received third generationcephalosporins such as ceftriaxone followed by second-generation cephalosporins such as cefuroxime (Haney et al., 2020). In our study also, ceftriaxone was more commonly used than cefuroxime. Wound classification, the timing of pre-surgical antimicrobials, or surgery sites were preferring ceftriaxone over cefuroxime or vice versa. In a study related to orthopedic surgeries, ceftriaxone and cefuroxime were their choices of antimicrobials (Dhammi et al., 2015). The antimicrobial choice for surgeries related to the breast was ampicillin/ampicillin-sulbactam and third generationcephalosporins (Bağhaki et al., 2014). From our study, within the use of cefuroxime, it was preferred more in orthopedic and ENT surgeries though less than ceftriaxone. ASP of the hospital shall focus first on these surgeries to improve the use of cefuroxime.

Prescribing post-surgical antimicrobials at discharge from the hospital were common at the study site. In many of these cases, ASHP guidelines recommend against the need for antimicrobials. Considering the environmental factors, the ability of patients for their self-care, misuse of antimicrobials shall be justified, but it is important to consider a decrease in the use of antimicrobials. In clean and clean-contaminated surgeries, hospital discharge antimicrobials shall be avoided if there is no specific indication in a particular patient. Misuse of antimicrobials is a global problem that needs to be addressed (Loozen et al., 2017; MondeloGarcía et al., 2018; Podda et al., 2019; Zaha et al., 2020). Health insurance not covering expenses of SSIs and patient preferences to take over precaution might be contributing to overuse of antimicrobials at the study site. 

This tertiary-level care hospital has an established ASP. Which has a policy to prescribe the antibiotic as surgical prophylaxis to different classes of surgeries. This policy is supported by IDSA & SEHA guidelines and it promotes cefuroxime for clean & clean-contaminated cases. But the reason for not compliance to the policies being many like; 1) Change of surgeons, new surgeons need time and motivation to change old habits of prescribing. 2) the surgeons feel comfortable to start with ceftrixone as prophylactic and continue as therapeutic antibiotic until the patient gets discharge. 3) Non-availability of cefuroxime for some period of the year. 4) pressure from insurance companies for early discharge post-operative and surgeons feels ceftrixone is superior and broad spectrum antibiotic.

After this study, the Antibiotic Stewardship Committee shall discuss this presentation and highlights the significance & effectiveness of cefuroxime as pre-surgrical prophylaxis. After a period of 1 year, a comparative analysis can be presented showing that the number % of surgical site infections as remained to minimal so as when ceftriaxone was prescribing.

## Limitations

The study has limitations regarding its retrospective design. Some data were missing, especially about intraoperative antimicrobial use. The accuracy of data might also be compromised compared to a prospective design. Still, a retrospective design was used, considering the feasibility of the study. For the data collected from medical records, there is the possibility that some of the antimicrobials were used for the treatment of infections instead of prophylaxis of SSIs. Health insurance not paying for the management of SSIs might add pressure to overuse antimicrobials.

Another limitation of the study is whether any patients had some level of immunosuppression that was not mentioned in the medical records. In such patients, more than the usual use of antimicrobials shall be justified. We did exclude patients who were having a transplant, cancer, or consuming any immunosuppressant drugs. Collecting data from cesarean sessions might be useful as a separate study as they were high in number. Though the current number of surgeries described a pattern in antimicrobial use, more data will help generate more hypotheses to be tested. The current analytical cross-sectional design was used to generate a hypothesis only, not to test it.

## Clinical Hypothesis

The surgical site showed association to the choice of ceftriaxone. In all cases, ceftriaxone was preferred over cefuroxime based on descriptive data. The following hypothesis is generated to be tested with adequate sample size, “ceftriaxone is not preferred over cefuroxime in surgical antimicrobial prophylaxis of surgeries categorized based on sites of surgeries.” While discharging patients’ home after surgery, unnecessary use of antimicrobials was also observed.

## Conclusion

The study showed the overuse of antimicrobials in surgical prophylaxis. In dirty surgeries, higher use of antimicrobials is essential as it treats the existing infection. In other cases, overuse of higher classes of antimicrobials is observed in SAP. No surgical site infection happened among any of the study population. In relation to ASHP SAP guidelines, overuse of antimicrobials was found before and after surgery and at the discharge of the patients. The selection of antimicrobials was also different compared to the ASHP guidelines and the guidelines at the study site. Instead of first or secondgeneration cephalosporins, ceftriaxone (third generation) was more commonly used. A comparative analysis is recommended after a period of 1 year to review the antibiotic prescribing and rate of surgical site infections.

## Data Availability

The original contributions presented in the study are included in the article/Supplementary Material, further inquiries can be directed to the corresponding author.
